# Bending energy of 2D materials: graphene, MoS_2_ and imogolite†

**DOI:** 10.1039/c7ra10983k

**Published:** 2018-01-25

**Authors:** Rafael I. González, Felipe J. Valencia, José Rogan, Juan Alejandro Valdivia, Jorge Sofo, Miguel Kiwi, Francisco Munoz

**Affiliations:** Centro de Nanotecnología Aplicada, Facultad de Ciencias, Universidad Mayor Santiago Chile rafael.gonzalezvaldes@mayor.cl; Centro para el Desarrollo de la Nanociencia y la Nanotecnología (CEDENNA) Santiago Chile; Departamento de Física, Facultad de Ciencias, Universidad de Chile Casilla 653 Santiago Chile; Núcleo de Matemáticas, Física y Estadítica, Facultad de Ciencias, Universidad Mayor Manuel Montt 367, Providencia Santiago Chile; Department of Physics and Material Research Institute, The Pennsylvania State University, University Park Pennsylvania 16802 USA

## Abstract

The bending process of 2D materials, subject to an external force, is investigated, and applied to graphene, molybdenum disulphide (MoS_2_), and imogolite. For graphene we obtained 3.43 eV Å^2^ per atom for the bending modulus, which is in good agreement with the literature. We found that MoS_2_ is ∼11 times harder to bend than graphene, and has a bandgap variation of ∼1 eV as a function of curvature. Finally, we also used this strategy to study aluminosilicate nanotubes (imogolite) which, in contrast to graphene and MoS_2_, present an energy minimum for a finite curvature radius. Roof tile shaped imogolite precursors turn out to be stable, and thus are expected to be created during imogolite synthesis, as predicted to occur by self-assembly theory.

## Abstract 2

The bending process of 2D materials, subject to an external force, is investigated, and applied to graphene, molybdenum disulphide (MoS_2_), and imogolite.

## Graphical abstract

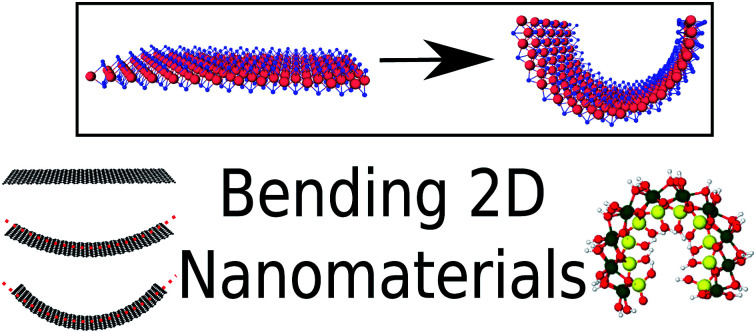

## 1 Introduction

One of the most exciting possibilities of nanotechnology is to control electronic properties at the atomic level, since new degrees of freedom become available, as compared to bulk materials. Among them, applying out-of-plane strain is a very versatile way to engineer several properties. In two-dimensional (2D) van der Waals materials, the strain is very anisotropic, and the folding and bending has recently attracted particular attention.^1–5^ Even the possibility of nano-kirigami has been explored, and stretchable transistors have been fabricated.^6,7^

Among the systems where the bending of a graphene sheet is relevant, an example is the rippling of suspended graphene with mesoscopic amplitudes and wavelengths.^8^ Also, supported graphene monolayers have been intensely studied.^9^ The effect of different metallic substrates has been investigated as well, showing that roughness can be tuned by varying the support.^10^ Recently, Zhou *et al.*^11^ reported on the interaction with a substrate where graphene forms wrinkles and also adopts different conformations. Theoretical studies have shown that the bending stiffness of a graphene monolayer is critical to achieve structural stability, and it determines the morphology for both suspended and supported graphene sheets.^12^ Moreover, it could have significant effects on their electronic properties.

Other 2D materials like h-BN, MoS_2_ or WSe_2_ have also been the focus of attention, since the control of the roughness, or the use of wrinkles, favors some applications. Moreover, new stable 2D materials and their hetero-structures^13^ are being synthesized or proposed at a fast pace. Therefore it is highly desirable to have simple models that are able to describe their basic properties. However, models need parameters in order to distinguish among the different materials.

The main objective of this work is to implement a strategy to calculate the energy that is needed to bend 2D materials (*i.e.* to calculate the bending modulus). This strategy does not rely on a specific theory level or code. It uses an external force, specifically a harmonic potential with the desired geometry, that imposes a specific energy minimum. While we use a cylindrical geometry, other configurations such as a horseshoe are also possible. Once the harmonic potential is turned on, the system is allowed to relax and to adopt the desired shape, but it remains free of any additional constraint or strain.

Other strategies to study the bending response of 2D and layered materials have been put forward. For example, the bending energy of graphene has been studied for carbon nanotubes of different radii.^14–17^ Recently Xiong *et al.* computed the response of a MoS_2_ single layer^18^ with a method that applied external forces to the edges of a MoS_2_ sheet. Another approach uses a bent carbon template to control the curvature of aluminosilicates, such as a montmorillonite layer.^19^

We start with a simple case – graphene – which allows us to test our method against available data.^14–16^ Next, we apply the method to a MoS_2_ layer; that is, we generalize to a two atomic species system three atoms thick, coupling classical potentials with DFT, in order to study the effects of bending on the electronic structure, at a low computational cost. Finally, we study the bending of imogolite, a versatile aluminosilicate material that spontaneously adopts a nanotube structure. Imogolite has four atomic species, and is ≈0.5 nm thick. In contrast to graphene and MoS_2_, the bending energy of imogolite has a minimum as a function of diameter, and is highly monodisperse (*i.e.* quite uniform in diameter).^20–24^

Two hypotheses on the formation of imogolite have been put forward, and they are subtly different; however, in both the bending energy plays a key role. The hypothesis by Maillet *et al.*^25^ models the growth of imogolite in two stages. During the first one, the transformation of roof tile shaped protoimogolite into short nanotubes takes place. In the second stage the tip–tip coupling of these short nanotubes allows for the nanotube growth. The other hypothesis was presented by Yucelen *et al.*^26^ and suggests the self-assembly of protoimogolite to form the nanotubes. While both theories imply the occurrence of protoimogolite, only the former considers an intermediate short nanotube stage. Here we show that curved pieces of imogolite could exist and that they are energetically stable, supporting the plausibility of both hypotheses.

## 2 Methods and model

### 2.1 Bending model

**Fig. 1:**
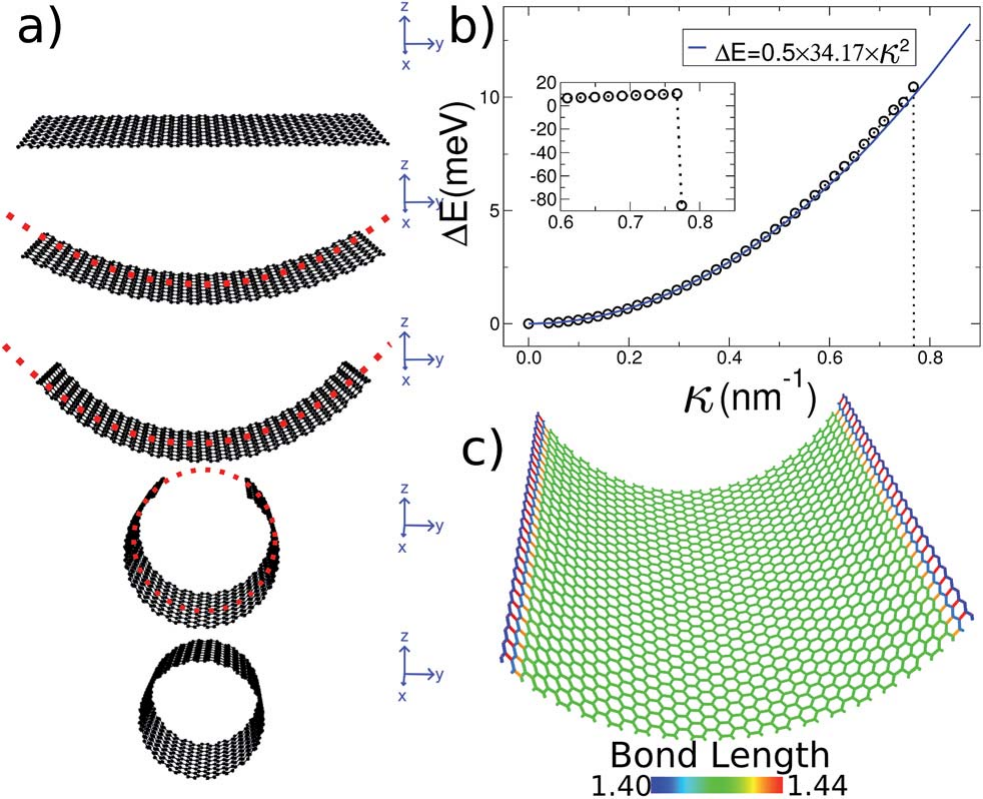
General procedure for our simulations. (a) Starting with a flat ribbon (*e.g.* graphene), an external potential is applied to smoothly generate the desired shape. The only strain source is the external force. (b) Strain energy Δ*E*(*κ*) for several curvature radii. The inset shows the abrupt energy change as the nanotube closes. (c) All of the carbon bonds are colored according to their length, showing that the 2D structure is maintained during the whole bending process.

A 2D sheet subject to a cylindrically symmetrical harmonic external potential adopts a curvature imposed by the external force, as illustrated in Fig. 1a. In this paper we study three different systems: graphene, MoS_2_ and imogolite sheets. Also, to show that this strategy is general enough, we carried out density functional theory (DFT) calculations, as implemented in the VASP code,^27–30^ for graphene and MoS_2_.

Our initial graphene configuration is a flat ribbon, centered at the origin (which corresponds to the harmonic potential being set equal to zero, as illustrated in Fig. 1a); next we allow the system to relax. Thereafter, we adopt (in small steps) finite values for the curvature radius, and relax again; this procedure is iterated until the smallest possible *R* is reached (*i.e.* the nanotube geometry). Throughout this paper we use periodic boundary conditions along the long edge of the ribbon in order to simulate an infinitely long system. During this process the 2D structure of graphene is preserved without inducing strain on the system. In fact, in Fig. 1c we show that during the bending process the bond length of the carbon atoms is kept around the equilibrium distance.

The energy associated to the external force is1
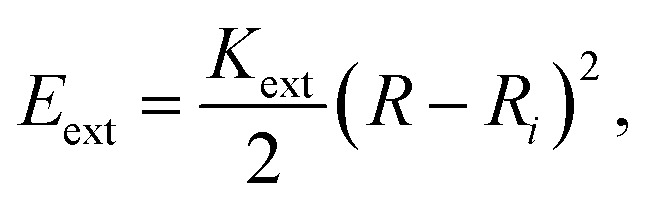
where *K*_ext_ = 250 eV Å^−1^, *R* is the curvature radius, and *R*_*i*_ is the distance of each atom, subject to the external force, to the center of curvature. On the other hand, the strain energy of the system, as a function of the curvature *R*^−1^ = *κ*, is given by2
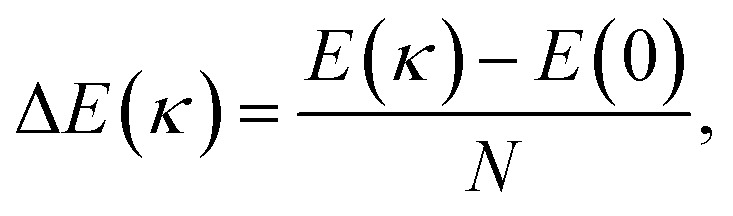
where *E* is the total potential energy of the system (ignoring the external energy, *E*_ext_), *N* is the number of atoms and *κ* is the curvature imposed by the external force (eqn (1), see Fig. 1b). The bending modulus^16^*C*_b_ is defined as the second derivative of the strain energy with respect to the curvature *κ*3
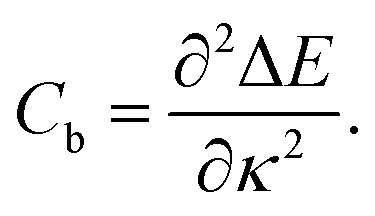


### 2.2 Computational methods

In the study of the bending process we used classical molecular dynamics simulations, as implemented in the large-scale atomic/molecular massively parallel simulator (LAMMPS) code.^31^ The bending due to the external force was combined with energy optimization strategies. We used the fast inertial relaxation engine^32^ (FIRE) together with conjugate gradient minimization. We chose this combination because the FIRE is able to avoid getting stuck in local minimum energy configurations, while the conjugate gradient is faster.

The graphene C–C atomic interactions were modeled by the ReaxFF force field.^33^ The ReaxFF parameters for the C–C interactions were first generated by Chenoweth *et al.*^34^ and later improved by Srinivasan *et al.*^35^ This force field has been tested for different carbon allotropes such as fullerenes, graphene, carbon nanotubes, amorphous carbon and diamond, with very good results.^35–38^ The MoS_2_ sheet was modeled with a second-generation reactive bond-order formalism by Liang *et al.*,^39,40^ and has been used to investigate the mechanical properties of MoS_2_ nanotubes,^41^ nano-indentation of crystalline MoS_2_,^42^ for a few layers of MoS_2_ under tension,^43^ and for the properties of a MoS_2_ layer.^18,44–47^ Finally, in order to model imogolite atoms we used the CLAYFF potential,^48^ since it has been proven to be adequate to model this aluminosilicate nanotube.^20,21,49,50^ The CLAYFF potential, developed by Cygan *et al.*,^48^ incorporates the charges of every single atom, the van der Waals interactions, a harmonic potential for the O–H group stretching, and harmonic stretching for the angles between Al–O–H and Si–O–H.

The band structure calculations were obtained with the VASP package.^27–30^ PAW pseudopotentials^51,52^ were used and the PBE exchange correlation^53^ was employed. The energy cutoff was set to 400 eV for graphene and to 300 eV for MoS_2_. We used 20 *K*-points along the only periodic direction. At least 15 Å of the vacuum were included in the non-periodic directions. The open visualization tool (OVITO) was used^54^ for the graphics and for the post-processing of our simulations, and PyProcar was employed to perform the band structure analysis.^55^

## 3 Results and discussion

### 3.1 Graphene

The initial configuration for our simulations is a graphene ribbon with periodic boundary conditions along the bending axis (*i.e.* perpendicular to the external forces) which is 8 nm wide. In Fig. 1a we illustrate the general procedure for our simulations of the ribbon bending, with dangling bonds and zig-zag termination. We represent the minimum of the external potential imposed for each curvature as a red dashed line. As shown in Fig. 1b the energy increases as a function of curvature *κ*, as expected. This behavior of the graphene strain energy is well known. Since a graphene sheet has dangling bonds at the edges there is an abrupt decrease in the total energy when the system finally adopts the nanotube geometry (*κ* ≈ 0.78 nm^−1^). To quantify the effect of the external force on the ribbon geometry in Fig. 1c we plot the pair correlation function *g*(*r*) for the three stages of the bending process. These stages are: the beginning, where the sheet is completely flat; an intermediate curvature; and finally as a CNT, when we turn off the external force and allow the system to relax. During the whole process the *g*(*r*) peaks associated to the first, second and third neighbors are located at 1.42, 2.46 and 2.84 Å, respectively. This constitutes evidence that the external force preserves the 2-D nature of the system during the whole process. Some smaller peaks, observed during the bending stage, are associated with the relaxation of the free edges, and disappear when the CNT geometry is achieved, when all the C atoms are sp^2^ coordinated.

**Fig. 2:**
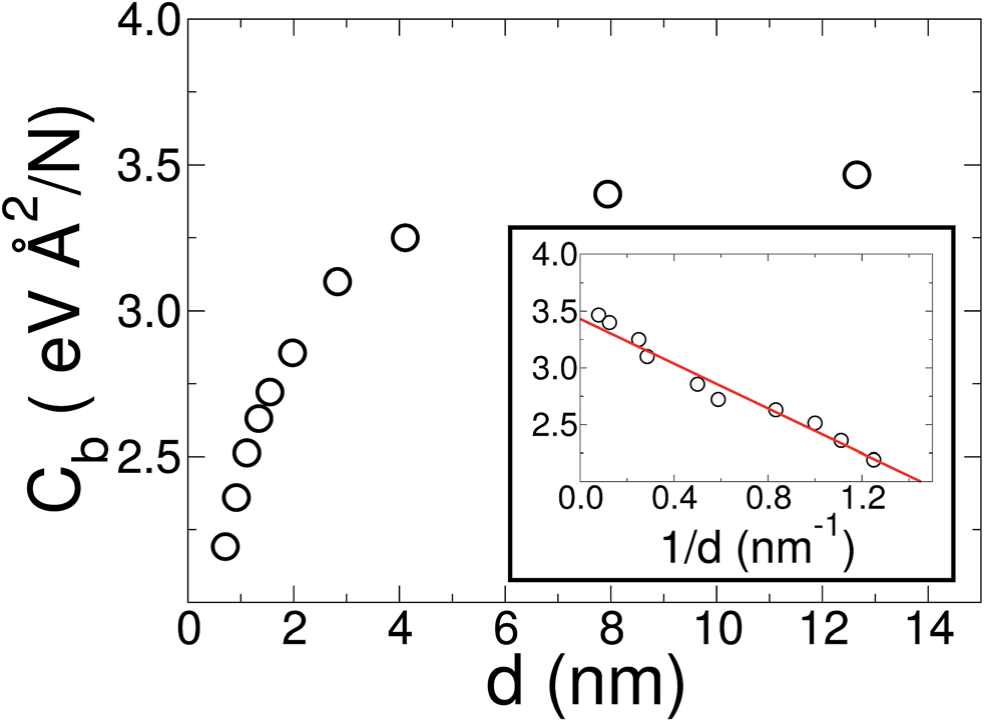
Bending modulus of a graphene ribbon as a function of its width *d*. While the usual methods, such as indentation or CNT bending energy, ignore finite size and edge effects, our method captures them. On the other hand, in the large diameter limit, where edges are negligible, these effects can be ignored. In the inset, we show a linear regression of the bending modulus as a function of 1/*d* (solid red line). From this linear regression we estimate a value of 3.43 eV Å^2^ per atom for graphene (1/*d* → 0 limit).

In Fig. 1b we fitted a quadratic curve, of the form 
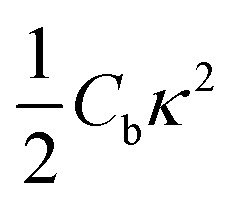
, for the 8 nm wide ribbon, obtaining a value for the bending modulus of 34.2 meV nm^2^ per atom = 3.42 eV Å^2^ per atom. However, for a narrow width ribbon finite size effects can be important, and have to be included. In fact, in Fig. 2 we observe that for a 0.7 nm ribbon the *C*_b_ is around 65% of the 8 nm value; but, for a 4 nm ribbon the *C*_b_ is already ≈95% of the 8 nm value. In other words, ribbons less than ∼2.5 nm wide are substantially easier to fold.

To obtain the *C*_b_ value for graphene we adopt the result obtained by the linear regression shown as an inset in Fig. 2, which corresponds to 3.43 eV Å^2^ per atom. This value is closer to the DFT values^14,15^ of 3.9 and 4.3 eV Å^2^ per atom than the classically calculated ones of 1.8 and 2.2 eV Å^2^ per atom.^16^

#### 3.1.1 Changes in electronic properties due to bending

**Fig. 3:**
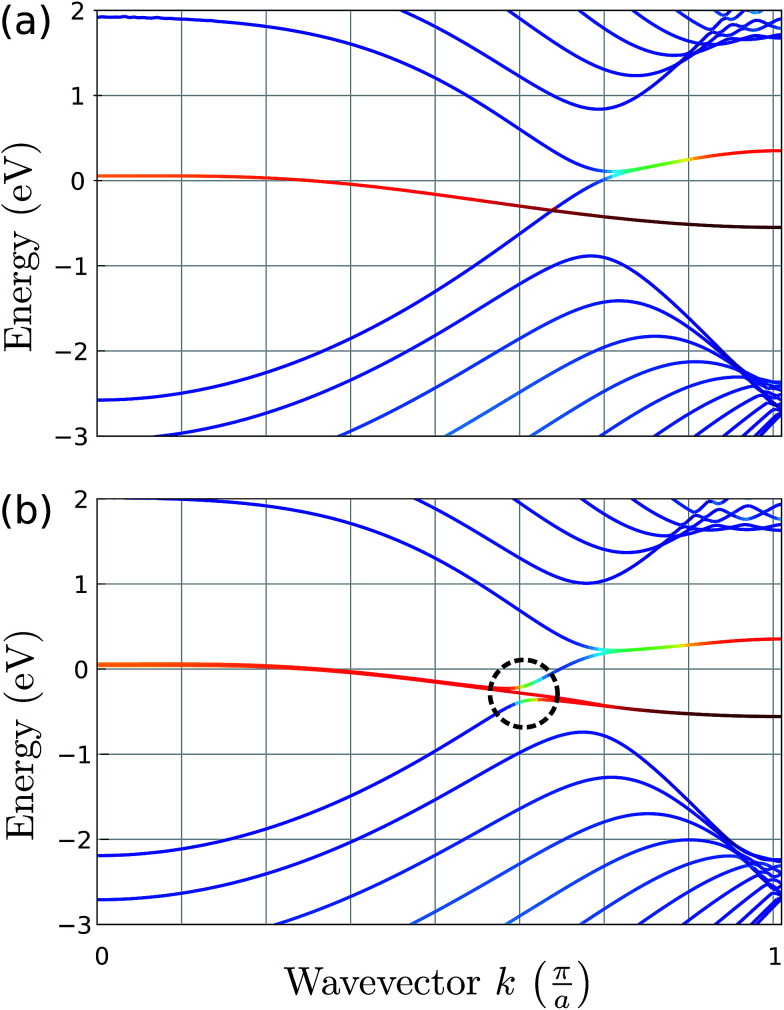
Band structure of a flat GNR (a) and a bent GNR, with the curvature radius *R* = 0.5 nm (b). The reddish bands are derived from the edges of the ribbon, the blue bands do not have a projection on the edges and intermediate colors apply for mixed states.

To study the effects of bending acting upon a graphene nanoribbon (GNR), we employed DFT. We found an almost undistorted electronic structure of a bent zig-zag GNR, even for a very small curvature radius. In Fig. 3 we calculate the band structure of a zig-zag GNR with a thickness of 2.4 nm. The pristine and bent cases, Fig. 3a and b, look almost identical: a textbook projection of the graphene’s Brillouin zone on the GNR reciprocal space (the sub-bands already resemble graphene’s Dirac cone). The valence and conduction bands are degenerated from 
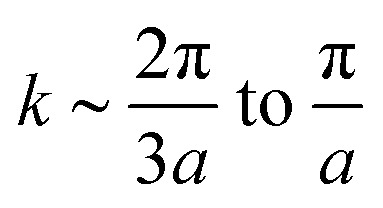
,^56^ due to the breaking of the sub-lattice symmetry at the boundaries. Additionally, there are flat bands, typical of the dangling bonds of terminal C atoms (they would disappear after hydrogenation). These dangling bonds are somewhat hybridized with bulk states for the bent GNR, which is the only noticeable difference compared to a flat GNR.

### 3.2 MoS_2_

Increasing the complexity of the 2D material under study, we now investigate the bending of a MoS_2_ ribbon. In this case we apply the bending force on the central Mo layer, while we allow the S atoms to move freely. It is worth remarking that the Mo atoms are able to move orthogonally to the constraining force, thus avoiding unwanted strain.

**Fig. 4:**
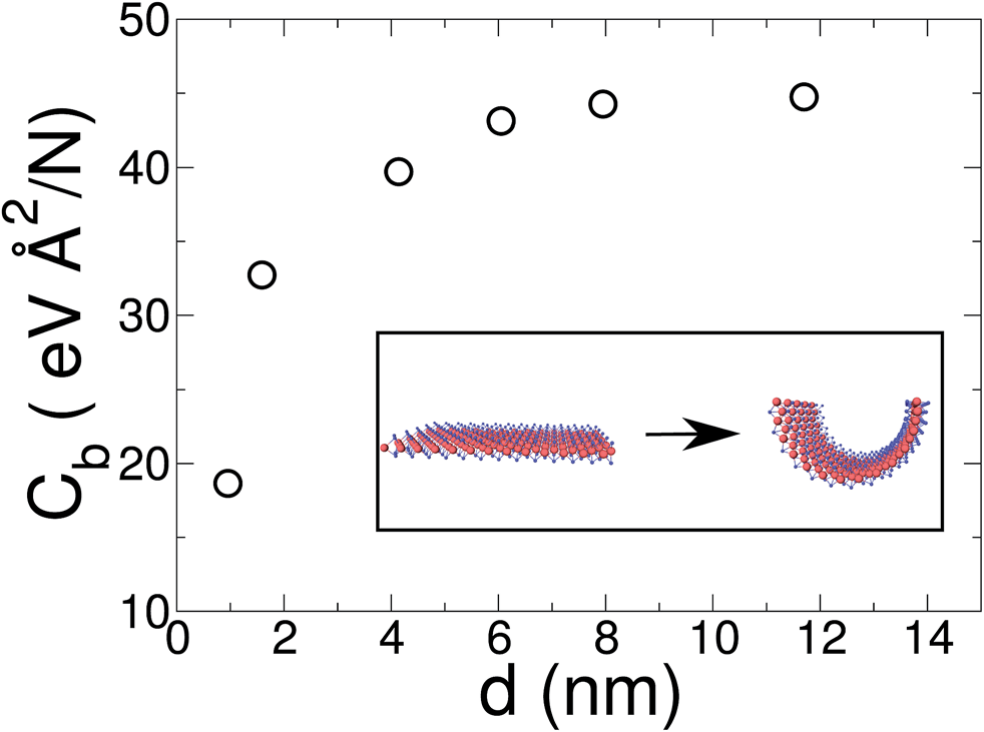
Bending modulus of a MoS_2_ ribbon as a function of its width *d*. MO: blue; S: red.

To compare the bending response of a MoS_2_ monolayer with the graphene case, we compute the bending stiffness^47^*D*, which is related to the bending modulus by *D* = *NC*_b_/*A*, where *A* is the transverse area of the ribbon. This way, the bending stiffness *D* of an 8 nm MoS_2_ ribbon turns out to be more than 11 times larger than that of a graphene ribbon of the same width. Additionally, we estimate the Föppl–von-Kármán number^6^ of a MoS_2_ monolayer to be about 40 times smaller than that of graphene. The Föppl–von-Kármán number is defined as *E*/*D*, where *E* corresponds to the Young modulus.^18,38^ Inspection of Fig. 4b of the paper by Blees *et al.*^6^ suggests that displacement, due to a laser, is negligible (less than the measurement noise). This argument does not rule out the feasibility of building nano-kirigami or foldable transistors^6,7^ but is an indication that the activation process would have to be different.

In a recent article Xiong and Cao compared DFT and molecular mechanics results for some MoS_2_ force fields.^47^ For the molecular mechanics they reported a bending stiffness of 8.7–13.4 eV depending on the force field used. On the other hand, they compared this with previous DFT results from Xiao *et al.* based on MoS_2_ single wall nanotube calculations.^57^ They have shown that the force field used in the present work slightly overestimates the bending stiffness with respect to the DFT results. Finally, using the values shown in Fig. 4, with a linear regression for 1/*d* → 0 limit, we obtain a bending stiffness for the MoS_2_ bulk layer of *D* = 16 eV. Casillas *et al.*^58^ reported a bending stiffness value of 4.1–13.24 eV, based on continuum theory. Our results for a MoS_2_ single layer are slightly over the values reported by other computational calculations and experiments.

In relation to the MoS_2_ finite width effects, in contrast with graphene ribbons, the difference between a narrow (0.95 nm) and a wide (8 nm) MoS_2_ monolayer is more pronounced. As can be seen in Fig. 4 the bending coefficient of our narrowest ribbon is only 42% of the value obtained for large sheets (*d* ≥ 8 nm).

#### 3.2.1 Electronic property changes due to bending

To obtain some insight into the changes in the electronic properties of a MoS_2_ ribbon due to curvature we employ first principles calculations. To alleviate the computational cost we used structures optimized classically (see previous section), but allowed the S atoms to relax; also, the strain in the periodic direction was minimized.

The (armchair) ribbon edges were not passivated, leaving dangling bonds. Eigenvalue analysis shows that these states remain highly localized at the edge of the ribbon – as expected of an insulating material – leaving bulk-like states in the inner region of the ribbon; therefore the exact details at the ribbon edges are not an issue. We will elaborate on this topic later on.

**Fig. 5:**
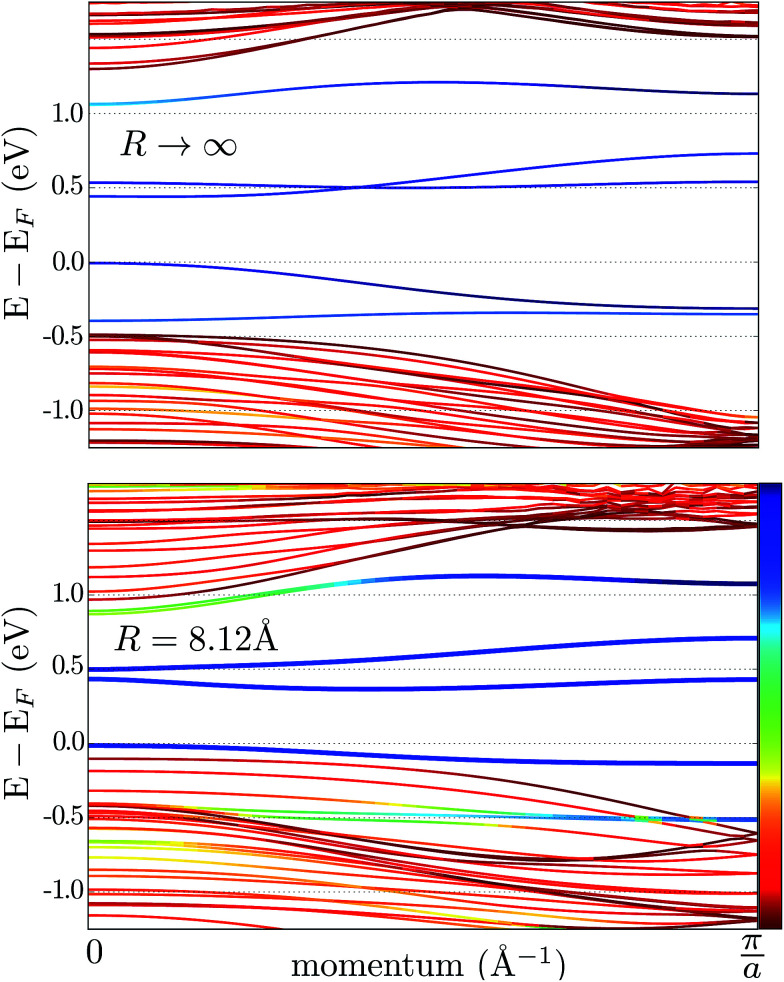
Band structure of an armchair MoS_2_ nanoribbon. The upper and lower panels correspond to a flat (*R* → ∞) and a bent nanoribbon, respectively. The color code shows the (real-space) localization of the eigenvalues: red is used for the inner subbands (*i.e.* bulk-like), while the edge subbands are blue; intermediate colors correspond to the hybridization of bulk and edge states.

**Fig. 6:**
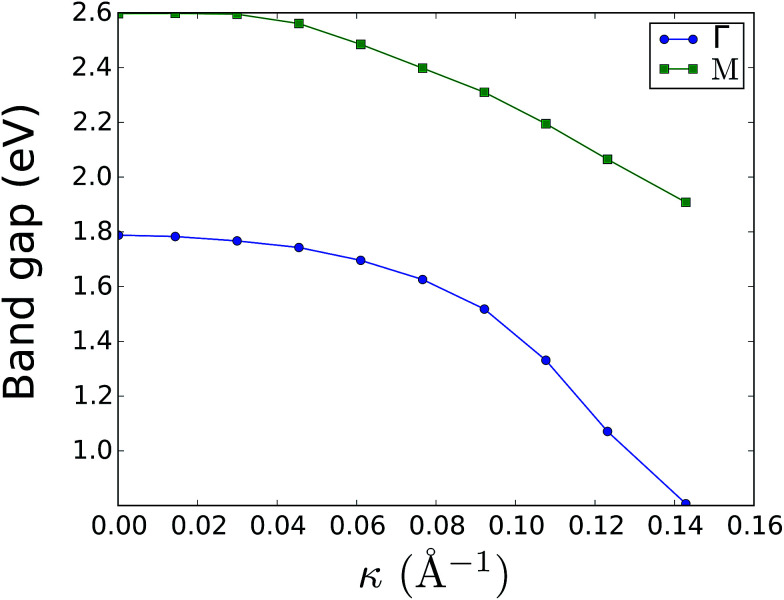
Electronic band gap at the *Γ* and *M* points as a function of curvature. The system band gap is always located at *Γ*; the band gap at *M* is just a parameter to quantify bending effects. Surface states were ignored in the gap measurement.

The effect of bending on the MoS_2_ band structure is shown in Fig. 5 for a flat and finite curvature radius system. It is evident that the inner, bulk-like bands (the red lines in Fig. 5) are qualitatively similar for *R* = 8.12 Å and *R* → ∞, but the gap between the valence and conduction bands is ∼0.8 eV smaller. Instead, the electronic states at the edge of the nanoribbon (blue in Fig. 5) suffer larger changes. This finding suggests that the bending effects on the electronic structure are rather modest; in fact, a bent MoS_2_ layer still behaves like flat MoS_2_ but with a smaller band gap, even for large curvatures. The surface states are likely to be more affected by the bending, but they remain localized at the edges. To quantify the extent of the change of the band-structure due to bending, Fig. 6 shows the band gap at two representative points (*Γ* and *M*) as a function of the curvature radius. Moderate or small bending results in small gap changes, while a tiny curvature radius (7 Å) can reduce the gap by ∼60%. We do not expect a metallic state to develop, or band inversion to occur, even for smaller radii. However, a more accurate calculation is required to obtain a precise quantitative picture.

### 3.3 Imogolite

**Fig. 7:**
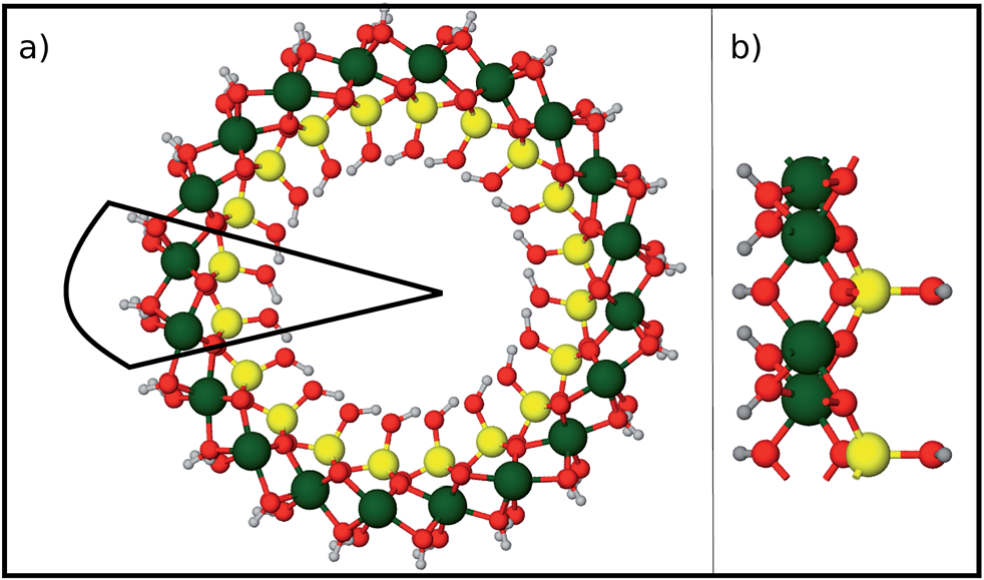
Structure of an imogolite nanotube. (a) An axial view of imogolite for *N*_*θ*_ = 10. The basic unit is marked by the continuous black line. (b) A side view of the basic imogolite unit [(OH)_3_Al_2_O_3_SiOH]_2_, that is repeated *N*_*θ*_ times to form the nanotube. Green Al; red O; yellow Si; and light gray H.

**Fig. 8:**
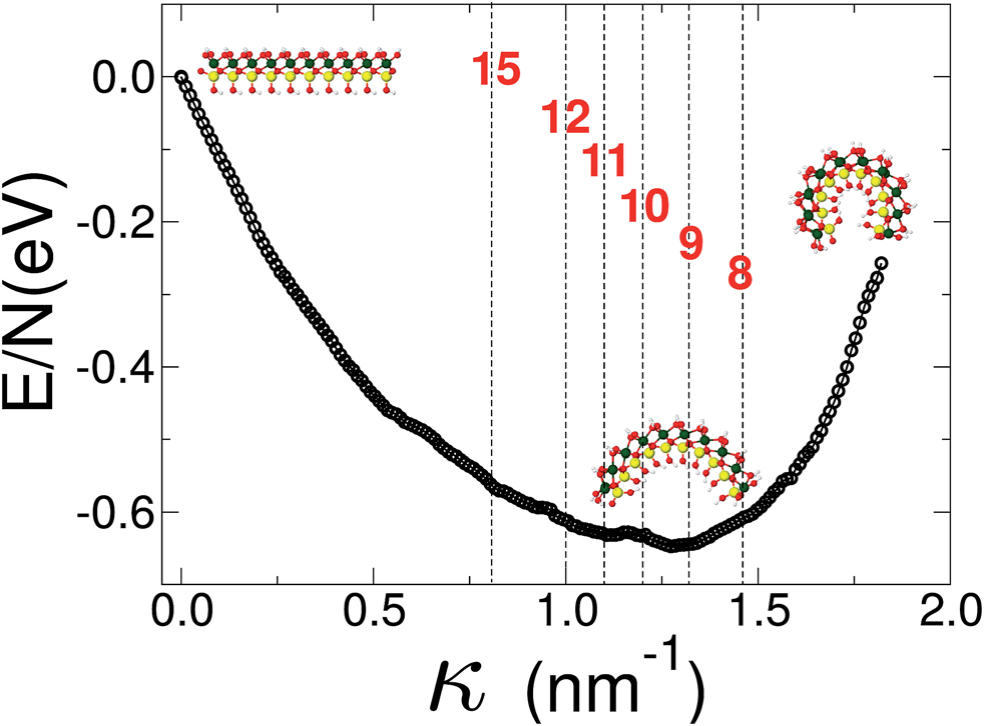
Energy *vs.* curvature of an imogolite sheet. The force is applied on the Al atoms. A minimum of the bending energy occurs for 9 < *N*_*θ*_ < 10. The imogolite structure inset corresponds to *N*_*θ*_ = 5 angular repetitions. The dashed vertical lines, and the numbers that label them, correspond to the curvature radius of a completely closed nanotube with that *N*_*θ*_ value. Similar data, for imogolite sheets for *N*_*θ*_ = 4 and 6, are included as ESI.† Green Al; red O; yellow Si; and light gray H.

Finally we report our results for the strain energy of a curved imogolite sheet. In Fig. 7 we provide on the left hand side an axial view of the imogolite tube, and on the right a view of its basic unit [(OH)_3_Al_2_O_3_SiOH]_2_. The single walled imogolite nanotube has the chemical composition [(OH)_3_Al_2_O_3_SiOH]_2*N*_*θ*__; this unit is repeated angularly, around the longitudinal axis, *N*_*θ*_ times, and *N*_*Z*_ times along the longitudinal direction.^59^ In Fig. 8 we show the energy of an imogolite sheet with this composition and *N*_*θ*_ = 5, *N*_*Z*_ = 6, assuming periodic boundary conditions along the longitudinal direction. The bending force is applied on the Al atoms. In Fig. 8 we include three insets of the sheet configuration: the initial planar sheet; the minimum energy configuration (half cylinder); and the structure obtained for the smallest curvature radius we calculated. Previously^21,49^ we showed that the energy minimum for an imogolite nanotube, using the CLAYFF force field, occurs for *N*_*θ*_ = 10. In the present case, a ribbon of half the width (*N*_*θ*_ = 5) reaches its minimum energy for a half-cylinder shape. We noticed that our results for the semi-cylinder indicate that it is energetically favorable as compared to forcing it to close upon itself to form a full cylinder. This reinforces the growth model proposed by Maillet *et al.*,^25^ suggesting that during the growth of imogolite, curved protoimogolite appear and assemble. The shallow minimum in Fig. 8 constitutes an indication that imogolite nanotubes of *N*_*θ*_ < 8 are energetically very unfavorable, due to the excess bending energy that is required. Moreover, this shallowness of the minimum also suggests that during the formation process, depending on the synthesis conditions,^26^ nanotubes 9 ≤ *N*_*θ*_ ≤ 15 can form, since the energy required is quite small.

## 4 Summary and conclusions

We studied the bending process of nano-sheets, by modeling and quantitatively treating the rolling of 2D materials into tubular conformations. To display the versatility and the wide range of applicability of the procedure we studied three different systems: graphene, MoS_2_ and imogolite. Moreover, our model is straightforward to generalize to other types of nano-sheets,^60^ like h-BN, MoSe_2_ and TiO_2_. It can also be applied to nano-sheets that roll around different kinds of nano-cylinder^61,62^ such as TiO_2_ nanotubes, metallic nanowires, CNTs (or MWCNTs) and imogolite-like nanotubes.^63,64^

For graphene (*κ* = 0) we obtained a bending modulus of 3.42 eV Å^2^ per atom, which is in agreement with the values in the literature.^16^ We also found that for thin graphene ribbons, edge effects can significantly decrease the magnitude of the bending modulus. This facilitates the graphene folding. We found that MoS_2_ is ∼11 times harder to bend than graphene. We also showed that it is possible to control the bandgap of a nano-sheet by bending it.

In conclusion, we have shown a strategy to compute the bending energy of 2D materials, which can be readily generalized to other 2D systems. It can also be used to predict the mechanical properties of novel heterostructures.^13^ In addition, we calculated how finite size effects, due to the presence of edge atoms, modify electronic and mechanical properties.

## Conflicts of interest

There are no conflicts to declare.

## Supplementary Material

RA-008-C7RA10983K-s001
