# Supplementary material for: Bending energy of 2D materials: graphene, MoS2 and imogolite
Source: RSC Adv. 2018 Jan 25;8(9):4577–83. doi: 10.1039/c7ra10983k (PMC9077804; doi:10.1039/c7ra10983k)
Supplement: RA-008-C7RA10983K-s001 [file RA-008-C7RA10983K-s001.pdf]

Electronic Supplementary Material (ESI) for RSC Advances.
This journal is © The Royal Society of Chemistry 2018

Electronic Supplementary Information: Bending Energy of 2D Materials: Graphene, MoS_2 and Imogolite

Rafael I. González,*[a,b], Felipe J. Valencia[b,c,d], José Rogan[b,c], Juan Alejandro Valdivia[b,c], Jorge Sofo[e], Miguel Kiwi[b,c] and Francisco Munoz[b,c]

[a] *Centro de Nanotecnología Aplicada, Facultad de Ciencias, Universidad Mayor, Santiago, Chile. E-mail: rafael.gonzalezvaldes@mayor.cl*
[b] *Centro para el Desarrollo de la Nanociencia y la Nanotecnología (CEDENNA) Santiago, Chile.*
[c] *Departamento de Física, Facultad de Ciencias, Universidad de Chile, Casilla 653, Santiago, Chile.*
[d] *Núcleo de Matemáticas, Física y Estadítica, Facultad de Ciencias, Universidad Mayor, Manuel Montt 367, Providencia, Santiago, Chile*
[e] *Department of Physics and Material Research Institute, The Pennsylvania State University, University Park, Pennsylvania 16802, USA*

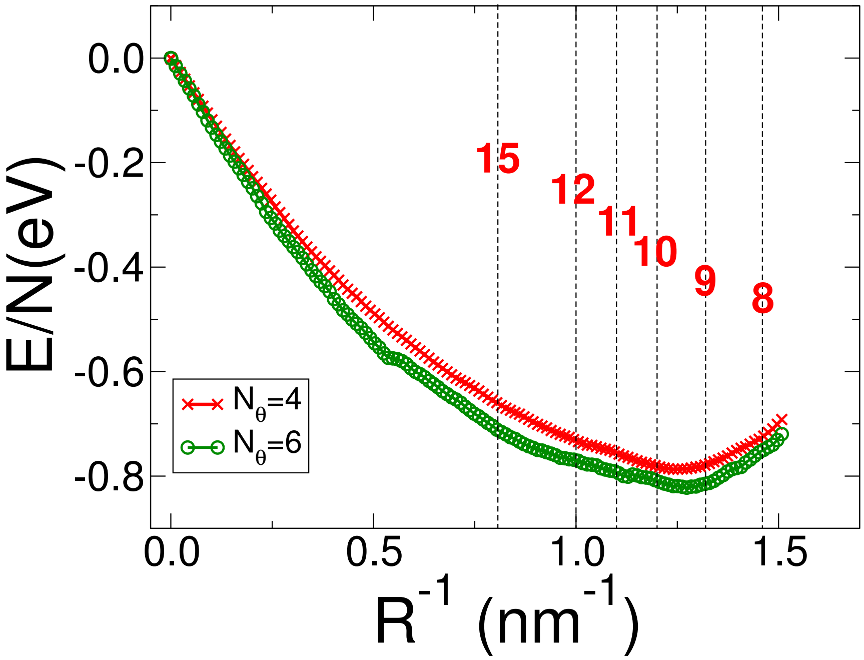

Figure S1: Energy vs. curvature of imogolite sheets with $N_\theta = 4$ and 6. For both cases the minimum of the bending energy occurs for $9 < N_\theta < 10$. The dashed vertical lines, and the numbers that label them, correspond to the curvature radius of a completely closed nanotube with that N_θ value. Green Al; red O; yellow Si; and light gray H.

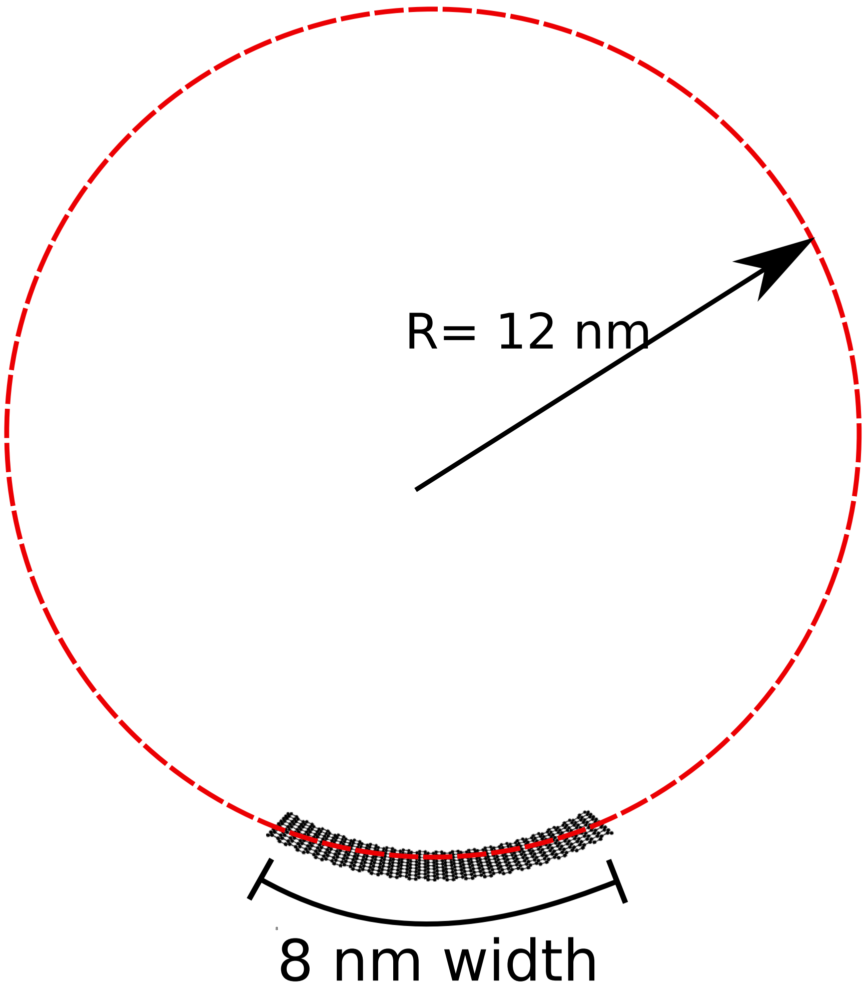

Figure S2: Illustration of the bending process: the external force has a curvature radii of 12 nm and is applied to a graphene ribbon 8 nm wide.
